# Vitamin D status in dogs with babesiosis

**DOI:** 10.4102/ojvr.v86i1.1644

**Published:** 2019-03-28

**Authors:** Eran Dvir, Chantal Rosa, Ian Handel, Richard J. Mellanby, Johan P. Schoeman

**Affiliations:** 1 Department of Companion Animal Clinical Studies, Faculty of Veterinary Science, University of Pretoria, Pretoria, South Africa; 2 Tel Hai Academic College, Upper Galilee, Israel; 3 Northwest Veterinary Specialists, Runcorn, United Kingdom; 4 Royal (Dick) School of Veterinary Studies and the Roslin Institute, University of Edinburgh, Scotland, United Kingdom

## Abstract

Canine babesiosis is a virulent infection of dogs in South Africa caused principally by *Babesia rossi*. Hypovitaminosis D has been reported in a wide range of infectious diseases in humans and dogs, and low vitamin D status has been associated with poor clinical outcomes. However, the relationship between vitamin D status and canine babesiosis has not been investigated. The objective of this study was to examine the relationship between the presence and severity of *B. rossi* infection and vitamin D status of infected dogs. Owners with dogs with a confirmed diagnosis of *B. rossi* infection and of healthy control dogs were invited to enrol onto the study. Vitamin D status was assessed by measurement of serum concentrations of the major circulating vitamin D metabolite, 25-hydroxyvitamin D (25[OH]D). Dogs with babesiosis (*n* = 34) had significantly lower mean serum 25(OH)D concentrations than healthy dogs (*n* = 24) (37.76 ± 21.25 vs. 74.2 ± 20.28 nmol/L). The effect of babesiosis on serum 25(OH)D concentrations was still significant after adjusting for any effect of age, body weight and sex. There was a negative relationship between serum 25(OH)D concentrations and disease severity in dogs with babesiosis. Serum concentrations of creatinine and alanine aminotransferase and time to last meal were not associated with serum 25(OH)D concentrations in dogs with babesiosis. In conclusion, dogs with *Babesia rossi* infections had lower serum 25(OH)D concentrations than healthy dogs. The inverse correlation between 25(OH)D concentrations and the clinical severity score indicate that hypovitaminosis D might be a helpful additional indicator of disease severity.

**Keywords:**

vitamin D; 25-hydroxyvitamin D; babesiosis; dog; *Babesia rossi*.

## Introduction

Babesiosis is a disease of considerable global significance that causes morbidity and mortality in humans and many domestic and wild animals, including dogs (Mellanby et al. 2011; Schoeman 2009). Canine babesiosis is characterised by a wide range of clinical signs including pallor related to the anaemic state and often icterus (Schoeman 2009). The clinical course can vary from chronic or subclinical through to peracute and fatal, depending on the virulence of the species and the susceptibility of the host (Bohm et al. 2006). Even in referral centres with access to advanced veterinary care, mortality rates range from 7% to 10% (Bohm et al. 2006; Schoeman et al. 2007). Consequently, there is a clear need to understand more about the factors involved in modulating the risk of babesia infections in dogs and to develop therapeutic approaches that improve patient outcomes.

The importance of vitamin D in maintaining skeletal health has been known for nearly a century (Elder & Bishop 2014). However, there is increasing interest in exploring the importance of vitamin D in regulating non-skeletal tissue following the discovery that a wide range of tissues, notably immune cells, can express the vitamin D receptor (Mellanby 2016; Provvedini et al. 1983). These observations resulted in numerous studies that have examined the relationship between vitamin D status, typically assessed by serum concentrations of the major vitamin D metabolite 25-hydroxyvitamin D (25[OH]D), and the development and outcome of a wide range of infectious diseases (Youssef et al. 2011). For example, a link has been made between vitamin D status and the development of mycobacteria, HIV and respiratory infections (Kearns et al. 2015). Studies in dogs and cats have similarly found a relationship between hypovitaminosis D and the presence of an infectious disease. Such observations include a study of *Spirocerca lupi* infections in dogs that found that infected dogs had a lower vitamin D status than healthy dogs (Rosa et al. 2013). In addition, cats with either mycobacteria or feline immunodeficiency virus (FIV) infections were also found to have lower 25(OH)D concentrations than healthy cats (Lalor et al. 2012; Titmarsh et al. 2015b).

Although there have been very few studies that have examined the relationship between vitamin D status and babesiosis in any species (Kules et al. 2014; Mert et al. 2009), numerous studies have linked low vitamin D status to both the development and outcome of infections in human and experimental models of malaria, a disease that has a similar pathophysiology to canine babesiosis (Luong & Nguyen 2015; Reyers et al. 1998). For example, plasma 25(OH)D concentrations were lower in children with severe malaria compared to healthy children (Cusick et al. 2014). Furthermore, supplementation with vitamin D ameliorated experimental cerebral malaria in mice (He et al. 2014). Lastly, the administration of vitamin D alongside arteether improved survival in mice with cerebral malaria compared to either agent used alone (Dwivedi et al. 2016). A proteomic approach revealed that vitamin D binding protein concentrations were lower in dogs with babesiosis, indicating that vitamin D metabolism may be altered in infected dogs (Kules et al. 2014).

The hypothesis of this study was that dogs with babesiosis would have a lower vitamin D status than healthy dogs. The aim of the study was to measure serum concentrations of 25(OH)D in healthy dogs and in dogs with confirmed babesiosis.

## Material and methods

### Selection of cases

This prospective study was performed on dogs with canine babesiosis presented to the Onderstepoort Veterinary Academic Hospital (OVAH) of the University of Pretoria in South Africa. An initial diagnosis was made upon admission and included detection of large *Babesia* spp. parasites on a stained thin capillary blood smear. Dogs were excluded from the study if they were less than 1 year old and had a history of previous exogenous corticosteroid therapy, known concurrent disease or *Ehrlichia canis* morulae detected on the blood smear. After admission the dogs were further excluded if concurrent disease was identified during their hospital stay or if their blood samples were positive for *Babesia vogeli* or *E. canis* by polymerase chain reaction (PCR) and reverse line blot (RLB) (Matjila et al. 2004). The PCR was conducted with a set of primers that amplified a 460–540 base pair fragment of the 18S small subunit (SSU) rRNA spanning the V4 region, a region conserved for *Babesia* and *Theileria*. The *Ehrlichia* PCR amplified the V1 hypervariable region of the 16S SSU rRNA (Bekker et al. 2002; Schouls et al. 1999). The membrane used for RLB included probes for *B. vogeli, Babesia rossi, B. canis* and *E. canis*. Twenty-four healthy dogs were used as the control group. These dogs were admitted to the OVAH for blood donation as part of the OVAH blood bank. They were considered healthy on the basis of a physical examination and routine laboratory testing (i.e. haematology and serum biochemical analysis).

### Study design

A physical examination was conducted, and history, including patient age, sex and duration of illness, was determined. Blood was collected upon admission and all dogs with babesiosis had a haematology and serum biochemistry analysis performed. Blood was taken from the jugular vein by needle venepuncture in all cases. Serum samples were allowed to clot and the tubes were spun down within 1 h. The serum was used for general diagnostic serum biochemistry and an additional tube was placed in dedicated plastic storage tubes and stored at -80 °C until 25(OH)D analysis.

The severity of the babesiosis cases was scored using the following parameters, with each criteria receiving a score of 1: basal serum cortisol > 275 nmol/L, basal plasma ACTH > 20 pg/mL, basal serum T4 < 2.8 nmol/L, serum albumin < 20 g/dL, serum potassium < 3.6 mmol/L, serum phosphate > 1.94 mmol/L, serum creatinine > 133 *µ*mol/L, 2, serum bile acids ≥ 55 *µ*mol/L, serum glucose < 3.3 mmol/L, presence of icterus, per cent of band cells of the total white cell count > 10% and body temperature < 37.8 °C (Bohm et al. 2006; Keller et al. 2004; Nel et al. 2004; Schoeman et al. 2007). Each case received an accumulated score (maximum 12).

In the healthy dogs, anamnesis, haematology, serum biochemistry, urinalysis and faecal analysis, including a modified centrifugal faecal flotation, were performed to rule out canine babesiosis or any other systemic disease. Exclusion criteria for all dogs of all groups included the following: less than 1 year of age, concurrent diseases, treated with medications that could influence vitamin D concentrations (corticosteroids, anticonvulsants, calcium channel blockers, diuretics). The appetite of all dogs diagnosed with babesiosis was recorded as time (in hours) since the last meal, based on information obtained from the owners.

Serum samples for 25(OH)D analysis were frozen within 1 hour of collection and stored at -80 °C prior to being sent to the laboratory for analysis on dry ice. Serum 25(OH)D concentrations were measured and validated as described in detail elsewhere (Gow et al. 2011; Mawer et al. 1990). Samples were extracted using acetonitrile and applied to C18 Silica Sep-Paks and metabolites separated by straight phase high-performance liquid chromatography. A Hewlett-Packard Zorbax-Sil column eluted with hexane:propan-2-ol:methanol (92:4:4) was used. Then serum 25(OH)D_2_ and 25(OH)D_3_ were measured separately by application to a second Zorbax-Sil, the column eluted with hexane:propan-2-ol (98:2) and quantified by UV absorbance at 265 nm (radioimmunoassay) and corrected for recovery (sensitivity 5 nmol/L, intra- and inter-assay coefficients of variation 3.0% and 4.2%, respectively). Results were expressed as total serum 25(OH)D concentrations. The Specialist Assay Laboratory CSB3 participates successfully in the vitamin D quality assurance scheme and is accredited to the International Organization for Standardization (ISO) 9001:2008 and ISO 13485:2003.

### Statistical analyses

General linear models were used to assess the relationship between variables of interest and 25(OH)D concentrations. Residual models were inspected for normality, heteroscedasticity and trend with other variables to assess model assumptions. Models were fitted using the R statistical system. A *p*-value of < 0.05 was considered statistically significant in all statistical tests performed.

### Ethical considerations

The dogs were only included in the study with owner consent. The study was reviewed and approved by the Animal Use and Care Committee of the University of Pretoria (Protocol V070-05).

## Results

**FIGURE 1:**
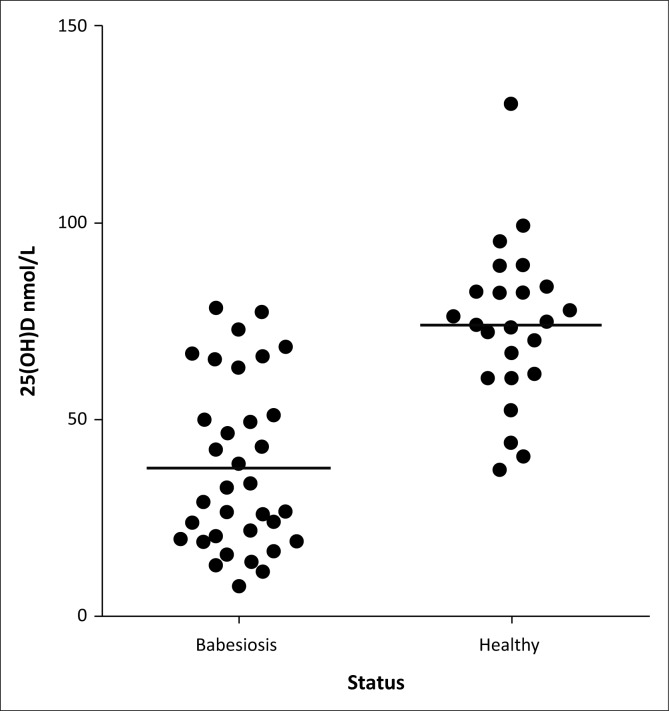
Serum 25-hydroxyvitamin D concentrations in dogs with babesia and healthy dogs. The black line indicates the mean concentration in each group.

Thirty-four dogs with babesiosis and 24 healthy control dogs were included in the study. Dogs included in both groups were of different breeds and more than 1 year of age. The common breeds in the babesiosis group were cross breed (10), Boerboels (4), German shepherd dogs (4), Fox terriers (3) and other breeds with ≤ 2 dogs. Their age ranged from 12 to 156 months (median 24 months). The common breeds in the control group were Beagles (5), Boerboels (4), Greyhounds (3), Fox terriers (3) and other breeds with ≤2 dogs. Their age ranged from 12 to 120 months (median = 30 months). The babesiosis group had significantly lower mean serum 25(OH)D concentrations than the control group (37.8 ± 21.3 vs. 74.2 ± 20.3 nmol/L; Figure 1). The effect of babesiosis on serum 25(OH)D concentrations was still significant after adjusting for any effect of age, body weight and sex (Table 1).

**TABLE 1:** Estimates from linear models of 25-hydroxyvitamin D concentrations exploring the relationship between vitamin D status and the presence of babesiosis, adjusting for other co-variables.

Variables	Estimate	SE	*t*	*p*
(Intercept)	55.62	6.75	8.24	< 0.0001
Age (months)	0.15	0.09	1.68	0.0970
Weight (kg)	0.35	0.18	1.95	0.0570
Babesiosis (reference = healthy control)	−35.93	5.59	−6.43	< 0.0001
Sex = male (reference = females)	7.29	5.69	1.28	0.2060

Note: The adjusted *R*^2^ value was 0.498.

SE, standard error; kg, kilogram.

The severity score of the babesiosis cases ranged from 0 to 10 with a median of 2. The negative relationship between serum 25(OH)D concentrations and disease severity was still significant after adjusting for any effect of age, body weight and sex (Table 2). The relationship between 25(OH)D concentrations in canine babesiosis and alanine aminotransferase (ALT), creatinine and time since last meal was explored in a linear model including additional co-variables of age, weight and sex. None of the clinical or biochemical parameters were associated with 25(OH)D concentrations in dogs with babesiosis.

**TABLE 2:** Estimates from linear models of 25-hydroxyvitamin D concentrations in dogs with babesiosis, exploring the relationship between vitamin D status and disease severity and adjusting for other co-variables.

Variables	Estimate	SE	*t*	*p*
(Intercept)	31.86	8.23	3.87	< 0.0001
Age (months)	0.34	0.23	1.48	0.1500
Weight (kg)	0.60	0.10	0.60	0.5600
Babesiosis (reference = healthy control)	−4.09	1.06	−3.86	< 0.0001
Sex = male (reference = females)	17.23	6.94	2.48	0.0200

Note: The adjusted *R*^2^ value was 0.356.

SE, standard error; kg, kilogram.

## Discussion

This study revealed that hypovitaminosis D is associated with canine babesiosis and that 25(OH)D concentrations are lower in patients with more severe disease. Reduced serum 25(OH)D concentrations could be attributed to anorexia or alterations in hepatic function. However, our analysis revealed that the decrease in 25(OH)D concentrations in dogs with babesiosis was independent of serum creatinine and ALT, thereby suggesting that the effect of renal insufficiency or liver damage was unlikely to be responsible for development of hypovitaminosis D. Moreover, our analysis revealed that hypovitaminosis D was not significantly correlated with the time from the last meal.

Our findings are consistent with previous studies, which have identified an association between hypovitaminosis D and the presence of an infectious disease and inflammation (Holowaychuk et al. 2012). Examples of recent studies showing this relationship are in dogs with leishmaniasis (Rodriguez-Cortes et al. 2017) and *S. lupi* infections (Rosa et al. 2013) and in cats with mycobacteriosis (Lalor et al. 2012) and FIV infection (Titmarsh et al. 2015b). The association between disease severity and vitamin D status identified in this study, with lower vitamin D concentrations associated with more severe disease, is also consistent with several previous studies in companion animals. Serum 25(OH)D concentrations were found to be negatively correlated to disease severity in studies of canine chronic enteropathies (Titmarsh et al. 2015a) and canine congestive heart failure (Osuga et al. 2015). The findings of our study differed from a smaller analysis of babesiosis in sheep, which found no difference in vitamin D status in infected and control animals (Mert et al. 2009).

The relationship between low vitamin D status and human malaria has also been extensively reported, with the pathophysiology of human malaria sharing many similarities with canine babesiosis (Luong & Nguyen 2015). Children with severe malaria were found to have significantly lower 25(OH)D concentrations than community children (Cusick et al. 2014). Despite the growing evidence that infectious diseases in companion animals and humans are associated with low vitamin D status, it still remains unclear whether the low serum 25(OH)D concentrations are the cause or consequence of the infectious state. A longitudinal study of 25(OH)D concentrations in humans with malaria found that vitamin D status did not change during the course of the infection, suggesting that 25(OH)D concentrations are unaffected during the evolution of an infectious process (Newens et al. 2006). The same could apply in canine babesiosis; however, additional research is required to more stringently examine whether low vitamin D status influences the risk of infectious disease development in both humans and companion animals. Moreover, the hypovitaminosis D seen in canine babesiosis could be because of an excess of vitamin D consumption during the inflammatory process resulting from a rapid conversion of 25(OH)D to the bioactive form 1,25-dihydroxyvitamin D by inflammatory cytokines (Autier et al. 2014). Alternatively, hypovitaminosis D could be present prior to babesiosis infections and predispose these dogs to disease development and increased disease severity. The time of the meal did not appear to influence the vitamin D results, making this hypothesis less likely in this study, but other factors involved in vitamin D storage and turnover affecting the serum 25(OH)D concentrations prior to disease could not be excluded. Ideally, the dietary vitamin D content should have also been evaluated in this study.

Hypovitaminosis D might have immunological consequences during the course of canine babesiosis infections, especially because vitamin D has a direct effect on the innate and adaptive immune response. The relationship between vitamin D status and immune function deserves further study in canine babesiosis, because it has been recently shown that dogs with canine babesiosis have alterations in their lymphocyte populations. A lower percentage of CD3+ and CD4+ lymphocytes were found in complicated babesiosis cases, and a lower percentage of CD8+ T lymphocytes was identified in babesiosis compared to healthy dogs, suggesting the presence of functional immune suppression (Rautenbach et al. 2017). We have previously reported that the active vitamin D metabolite, 1.25-dihydroxyvitamin D, can profoundly influence the phenotype and function of innate immune cells *ex vivo* (Besusso et al. 2015). Whether vitamin D status alters disease susceptibility and the evolution of the infection *in vivo* remains unclear.

In order to examine the relationship between vitamin D and infectious diseases, studies have investigated whether vitamin D supplementation can influence the outcome. In an experimental murine model of cerebral malaria, the co-administration of vitamin D alongside arteether was found to significantly improve survival. The improvement in outcome was associated with partially restored blood–brain barrier integrity and reduced serum pro-inflammatory cytokine concentrations (Dwivedi et al. 2016). Another study also reported that the oral administration of vitamin D, both before and after experimental malaria infection, protected mice from the development of cerebral malaria. There was reduced accumulation of pathogenic T cells in the brain and substantial improvement of the blood–brain barriers of malaria-infected mice in vitamin D–treated mice (He et al. 2014). Based on these observations, further studies exploring the role of vitamin D supplementation in canine babesiosis would be informative.

## Conclusion

We have shown that dogs with *B. rossi* infections have lower serum concentrations of 25(OH)D. Further studies are required to investigate whether vitamin D status plays a causative role in the development of babesiosis and whether improvement of vitamin D status can result in enhanced treatment outcomes. The inverse correlation between 25(OH)D concentrations and the clinical severity score indicate that hypovitaminosis D might be a helpful additional indicator of disease severity.
